# Impact of slice thickness on reproducibility of CT radiomic features of lung tumors

**DOI:** 10.12688/f1000research.141148.2

**Published:** 2023-12-01

**Authors:** Sanat Gupta, Kaushik Nayak, Saikiran Pendem

**Affiliations:** 1 Manipal College of Health Professions, Department of Medical Imaging Technology, Manipal Academy of Higher Education, Manipal, Karnataka, 576104, India

**Keywords:** Lung Cancer, Radiomics, Computed Tomography, Slice Thickness, CT Parameters

## Abstract

**Background:**

Radiomics posits that quantified characteristics from radiographic images reflect underlying pathophysiology. Lung cancer (LC) is one of the prevalent forms of cancer, causing mortality. Slice thickness (ST) of computed tomography (CT) images is a crucial factor influencing the generalizability of radiomic features (RF) in oncology. There is scarcity of research that how ST affects variability of RF in LC. The present study helps in identifying the specific RF categories affected by variations in ST and provides valuable insights for researchers and clinicians working with RF in the field of LC.Hence, aim of the study is to evaluate influence of ST on reproducibility of CT-RF for lung tumors.

**Methods:**

This is a prospective study, 32 patients with confirmed histopathological diagnosis of lung tumors were included. Contrast Enhanced CT (CECT) thorax was performed using a 128- Incisive CT (Philips Health Care). The image acquisition was performed with 5-mm and 2 mm STwas reconstructed retrospectively. RF were extracted from the CECT thorax images of both ST. We conducted a paired t-test to evaluate the disparity in RF between the two thicknesses. Lin’s Concordance Correlation Coefficient (CCC) was performed to identify the reproducibility of RF between the two thicknesses.

**Results:**

Out of 107 RF, 66 (61.6%) exhibited a statistically significant distinction (p<0.05) when comparing two ST and while 41 (38.3%) RF did not show significant distinction (p>0.05) between the two ST measurements. 29 features (CCC ≥ 0.90) showed excellent to moderate reproducibility, and 78 features (CCC ≤ 0.90) showed poor reproducibility. Among the 7 RF categories, the shape-based features (57.1%) showed the maximum reproducibility whereas NGTDM-based features showed negligible reproducibility.

**Conclusions:**

The ST had a notable impact on the majority of CT-RF of lung tumors. Shape based features (57.1%). First order (44.4%) features showed highest reproducibility compared to other RF categories.

## Introduction

Radiomics is a new field that seeks to improve the physician’s visual perception of medical images with addition of more quantitative objectivity. The quantitative attributes from radiographic images are utilized to characterize spatial and textural patterns of lesions which can provide information about the heterogeneity associated with biological processes. Radiomics is a rapidly evolving field particularly in oncology to improve patient care, aid in treatment decision making, characterization, response to therapy and prognosis. ^1^^–^^5^

Lung cancer/carcinoma (LC) remains one among the most prevalent and familiar types of cancer that results in mortality notwithstanding recent improvements in healthcare. As, most detected LC are in the middle to late phase of the disease progression and have few management options left, hence, people with lung cancer have a 10-20% survival rate at 5 years following the diagnosis in most of the developed nations. ^6^^,^^7^ Radiomics and Machine learning methods have been used for classification of histological subtypes of LC, prediction of LC staging and outcome, response to treatment, prognosis of lung cancer. ^8^^–^^11^

Radiomics, a rapidly evolving field, employs quantitative attributes from medical images to enhance physician’s interpretation, particularly in oncology. Radiomics and machine learning models developed based on radiomic features play crucial roles in classifying histological subtypes lung cancer. Evaluating the variability of radiomic features (RF) is important as diagnosis and treatment decision made using these quantitative should be precise and reproducible. Recent studies have shown that the CT technical parameters such as exposure factors, slice thickness (ST) and image reconstruction algorithms (IRA) can significantly affect the values of RF. Experts have recommended that for training predictive models using radiomics based machine learning models, only reproducible RF should be considered. ^12^^–^^14^ The reproducibility of texture analysis of lung tumors is unclear and there is scarcity of research that has delved into how ST affects variability of RF in lung tumors. The research gap of the study centers on the application of Radiomics in LC, emphasizing the need for a deeper understanding of the reproducibility of RF in the context of lung tumors. Hence, aim of the study is to evaluate the influence of ST on reproducibility of CT-RF for lung tumors.

## Methods

This is a prospective study. The study was commenced upon approval from the Institutional Ethical committee of Kasturba Medical College and Hospital, Manipal, India on 12 ^th^ August 2022 (IEC:193/2022) followed by the enrolment of the first subject after registration in the Clinical Trial Registry – India (CTRI) registration (CTRI/2022/09/045554) on 15 ^th^ September 2022, and continued till 30 ^th^ April 2023.

### Eligibility criteria

Patients with histopathological diagnosis of lung cancer types such as Non-Small Cell Lung Carcinoma (NSCLC) and Small Cell Lung Carcinoma (SCLC) were included. We excluded patients with ground glass nodules (GGN), lesions measuring less than 4mm, scans with motion artifacts and patients that did not consent to take part in the study. Ground glass nodules (GGN) often represent a distinct category of pulmonary nodules that may differ from solid lesions. Hence they were excluded to focus on a more homogenous sample. Limiting the inclusion criteria to lesions measuring at least 4mm ensures a more consistent and reliable measurement. Smaller lesions might present challenges in terms of accurate radiomic feature extraction and may be subject to greater variability due to partial volume effects, potentially influencing the study’s reproducibility findings. Written informed consent to participate was obtained from each patient.

### CT scanning procedure

The study was conducted at the Department of Radiodiagnosis, Kasturba Medical College and Hospital, Manipal, India. Both Kasturba Medical College and Hospital (KMC) and Manipal College of Health Professions (MCHP) are constituent colleges of Manipal Academy of Higher Education (MAHE). A total of thirty-two (32) patients with confirmed histopathological diagnosis of lung cancer (NSCLC- 71.8% & SCLC-28.1%) between September 2022 to April 2023 were included and all patients consented. The study population's demographic characteristics are outlined in Table 1.

**Table 1.** Demographic characteristics of study population.

Characteristics	Data
Age (Mean ± SD)	53.16 ± 10.25
Gender (%)	
Male (M)	18 (56.25 %)
Female (F)	14 (43.75%)
Tumor size, mm (Mean ± SD)	17.20 ± 15.77
Pathology (%)	Ca Lung/Pulmonary tumors • NSCLC-71.8% • SCLC-28.1%
Location (Lung)	
Right	10 (31.2%)
Left	7 (21.8 %)
Bilateral	15 (46.8%)

All patients underwent Contrast Enhanced CT (CECT) Thorax examination using 128 Slice Incisive CT (Philips Medical Systems). The protocol used for the CECT Thorax examination of the study population is detailed in Table 2. Retrospective reconstruction of the CT images was carried out utilizing CECT images from a standard protocol of 5mm to produce a ST of 2 mm. Contrast scans were performed using Iohexol 300 mgI/ml (General Electric Health care, Wisconsin, USA) as the contrast agent. The contrast media was administered using Dual Head CT Pressure injector, OptiVantage (Guerbet, France, UK).

**Table 2.** Technical parameters of CECT Thorax Protocol.

Protocol	Chest helical
Patient position	Supine - feet first
Scanogram	PA – 180 degree
Area coverage	Apex of lungs to the domes of diaphragm
Scan orientation	Craniocaudal
Acquiring Slice thickness	5mm
Slice increment	5mm
Kilovoltage (kVp)	120
Milliampere second (mAs)	360
Collimation	64 x 0.625
Rotation time	0.75 seconds
Field of view (FOV)	350 mm
Matrix size	512 x 512
Pitch	1.08
Reconstruction algorithm	iDose ^4^, Level 3
Spatial resolution	0.33
Contrast	Omnipaque – Iohexol (300 mgI/ml) Volume (60 ml)
Threshold (HU)	150
Window Width (WW)	400
Window Level (WL)	40

### Segmentation

**Figure 1.**
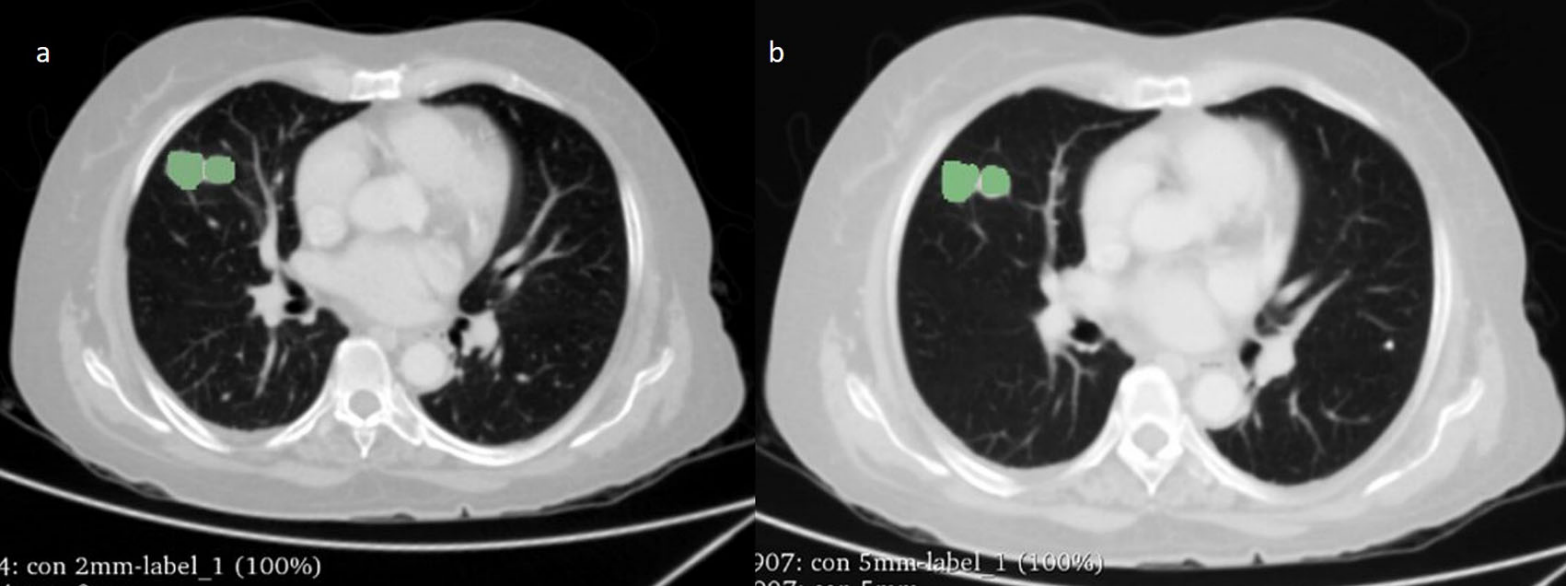
An axial CT image of a 52-year-old male with adenocarcinoma showing manual segmentation of tumour using 3D Slicer at (a) 2mm and (b) 5mm slice thickness.

The Digital Imaging and Communication in Medicine (DICOM) CECT sections of two different slice thickness (2 mm and 5 mm) of the same patient were loaded into 3D slicer (version 4.10.2) and a radiologist (Bharath J L) with over 10 years of experience manually delineated the tumours (see Figure 1 for an example). The segmentation was performed using lung window (Window Width (WW): 1500 HU and Window Level (WL): -600 HU). All pulmonary nodules/lesions present in the right, left, and bilateral lungs were segmented rather than solely selecting the largest or most prominent nodule/lesion. The segmentation of the nodule was performed while excluding the airways, blood vessels, or bronchi. We extracted RF from the segmented regions of the lung nodules using both 2-mm and 5-mm ST.

### Statistical analysis

Statistical analysis was done using SPSS version 20.0. A Paired t-test was performed to identify the significant difference in RF between the two slice thickness (2 mm and 5 mm) groups. Lin’s Concordance Correlation Coefficient (CCC) was calculated to assess the reproducibility of RF between two groups (2 and 5 mm). Concordance Correlation Coefficient of > 0.99 suggests excellent reproducibility, > 0.95 to 0.99 suggests good reproducibility, >0.90 to 0.95 suggests moderate reproducibility, ≤ 0.90 suggests weak reproducibility. p-value (<0.05) was considered.

## Results

A total of 32 cases (18 males and 14 females) with LC [Non-Small Cell Lung Cancer (NSCLC) – 71.8%, Small Cell Lung Cancer (SCLC) – 28.1%)] with mean age of were included 53.16 ± 10.25.

**Figure 2.**
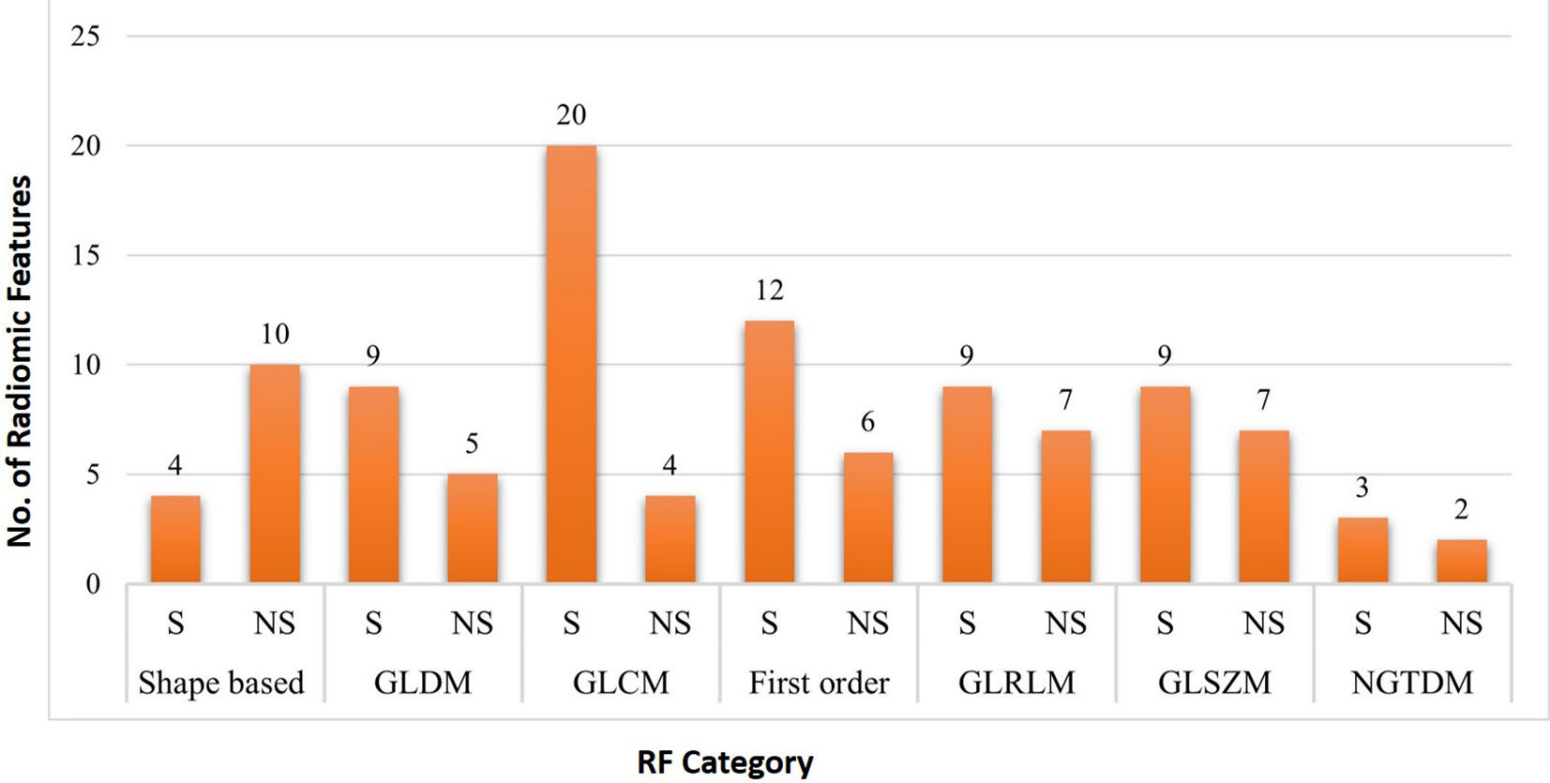
Number of significant (p<0.05) and non-significant (p>0.05) features in each RF category between 2-mm and 5-mm slice thickness.

A total of 3424 Radiomic Features (RF) measurements (107 RF per study) were extracted. Among them 66 (61.6%) RF exhibited significant difference between two the slice thickness measurements, while 41 (38.3%) RF did not show significant difference between the two slice thickness measurements ( Figure 2; Table 3).

**Table 3.** Percentage of significant and non-significant RF between 2-mm and 5-mm ST for each category.

RF Category	Features with p<0.05 [n(%)]	Features with p>0.05 [n(%)]
Shape (n=14)	4 (28.75%)	10 (71.42%)
GLDM (n=14)	9 (64.28%)	5 (35.71%)
GLCM (n=24)	20 (83.33%)	4 (16.66%)
First order (n=18)	12 (66.66%)	6 (33.33%)
GLRLM (n=16)	9 (56.25%)	7 (43.75%)
GLSZM (n=16)	9 (56.25%)	7 (43.75%)
NGTDM (n=5)	3 (60.00%)	2 (40.00%)

### Reproducibility of RF

**Figure 3.**
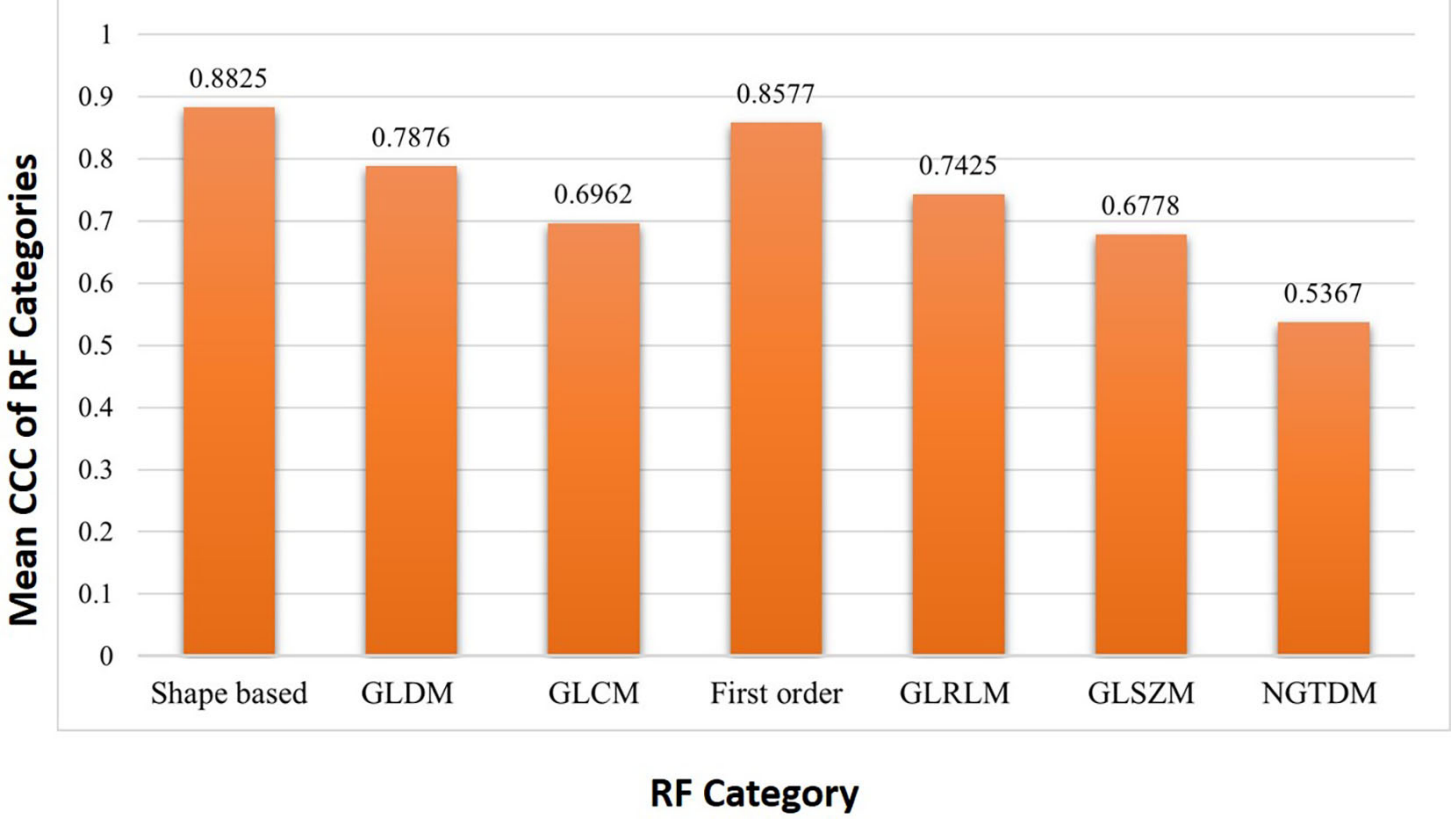
Mean concordance correlation coefficient (CCC) of each radiomic feature category between 2-mm and 5-mm slice thickness.

It was found that out of 14 shape-based features 8 (57.1%), out of 14 Gray Level Dependence Matrix (GLDM) RF 5 (35.71%), out of 24 for Gray Level Co-occurrence Matrix (GLCM) RF 3 (12.5%), out of 18 first order RF 8 (44.4%), out of 16 Gray level run length matrix (GLRLM) RF 4 (25%), out of 16 Gray level size zone matrix (GLSZM) RF 1 (6.25%) were found to be reproducible. All 5 neighboring gray tone difference matrix (NGTDM) RF were found to be not reproducible. Among the seven features categories, the shape-based features (57.1%) showed the maximum reproducibility whereas NGTDM based features showed negligible reproducibility ( Table 4). The mean CCC of RF categories were shown in Figure 3.

**Table 4.** Percentage of reproducibility of RF between 2-mm and 5-mm ST for each category.

RF Category	Excellent n(%)	Good n(%)	Moderate n(%)	Weak n(%)
Shape (n=14)	2 (14.2%)	3 (21.4%)	3 (21.4%)	6 (42.8%)
GLDM (n=14)	-	-	5 (35.7%)	9 (64.2 %)
GLCM (n=24)	-	-	3 (12.5%)	21 (87.5%)
First order (n=18)	-	1 (5.5%)	7 (38.8%)	10 (55.5%)
GLRLM (n=16)	-	1 (6.25 %)	3 (18.75 %)	12 (75 %)
GLSZM (n=16)	-	-	1 (6.25%)	15 (93.75%)
NGTDM (n=5)	-	-	-	5 (100%)

### Shape-based category

In shape-based category, features such as Voxel volume (0.997) and Mesh volume (0.997) showed excellent reproducibility. Major (0.973) and minor axis length (0.959), maximum 2D-diameter (0.976) had good reproducibility. Maximum 3D-diameter (0.944), maximum 2D-diameter slice (0.926) and maximum 2D-diameter row (0.903) had moderate reproducibility and rest of the six features showed poor reproducibility between 2- and 5-mm slice thickness.

### GLDM category

In GLDM category, features such as high gray level emphasis [HGLE] (0.918), dependence entropy [DE] (0.929), small dependence emphasis [SDE] (0.935), dependence non uniformity normalized [DNU] (0.935) and large dependence high gray level emphasis [LDHGLE] (0.903) showed moderate reproducibility and rest of the nine features showed poor reproducibility between 2- and 5-mm slice thickness.

### GLCM category

In GLCM category, features such as Idm (0.930), Id (0.922) and Sum squares (0.908) showed moderate reproducibility and rest of the twenty-one features showed poor reproducibility between 2- and 5-mm slice thickness.

### First order category

In first order category, features such as 10 ^th^ percentile (0.961) showed good reproducibility, Skewness (0.948), Uniformity (0.947), Median (0.921), Total energy (0.920), Root mean squared (0.945), Entropy (0.943) and Mean (0.943) showed moderate reproducibility and rest of the ten features showed poor reproducibility between 2- and 5-mm slice thickness.

### GLRLM category

In GLRLM category, features such as Gray level non uniformity normalized (0.952) showed good reproducibility, Short run emphasis (0.949), Run percentage (0.936) and Run length non uniformity normalized (0.943) showed moderate reproducibility and rest of the twelve features showed poor reproducibility between 2- and 5-mm slice thickness.

### GLSZM category

In GLSZM category, feature such as Zone percentage (0.906) showed moderate reproducibility and rest of the fifteen features showed poor reproducibility between two slice thicknesses.

### NGTDM category

In the NGTDM category, all the five features showed poor reproducibility between 2- and 5-mm slice thickness.

## Discussion

In the present study, we assessed the impact of slice thickness on the reproducibility of CT radiomic features (RF) for lung tumors. Few previous studies had addressed the influence of exposure parameters such as tube voltage (kV _P_), tube current (mA), image reconstruction algorithms (IRA), CT Scanner vendors on RF in CT for conditions like liver fibrosis, metastatic liver lesions, pancreatic neuroendocrine neoplasm. ^15^^–^^18^ Variability of acquisition parameters could affect the diagnostic performance of radiomic signatures in oncologic patients. ^18^^,^^19^ Limited studies had investigated the impact of ST on reproducibility of CT-RF in lung tumors.

In this study, the category of shape-based RF (57.1%) exhibited the highest reproducibility compared to other RF categories. These shape based features demonstrated robustness due to presence of low-frequency components and the reliance on segmented boundaries resulting in consistent reproducibility across changes in ST. Findings by Erdal et al. ^20^ & Lu et al. ^21^ supported this, revealing that RF describing tumor dimension, shape of boundaries, low-order density frequencies, and rough features were less sensitive to image setting parameters, in contrast to features characterizing sharpness of boundaries, high-order density frequencies and smooth features. Both studies analyzed the combination of ST with IRA (lung and standard) and noted that shape-based features were less effected by change in slice thickness and reconstruction algorithm. They also observed that the thinner slices with sharper reconstructions had fewer reproducible features compared to thicker slices with smoother reconstructions.

The GLDM category features in our study, such as HGLE, DE, SDE, DNU, LDHGLE demonstrated moderate reproducibility. A study by Emaminejad et al. ^22^ in non-contrast chest CT (NCCT) identified that GLDM DE, DNU, GLNU were reproducible against the dose and kernel variations with varying slice thickness. Unlike our study, none of the previous research mentioned the reproducibility of GLDM features concerning slice thickness alone.

Within the GLCM category in our study, only two features showed reproducibility with variations in slice thickness. Similar results were documented by Erdal et al. ^20^ & Kim et al. ^23^ indicating that GLCM category (19.4 % & 25 %) had lower reproducibility compared to other RF categories. We observed that first-order features (44.4%) had the second highest reproducibility. Studies by Erdal et al., ^20^ Park s et al., ^24^ Choe J et al. ^25^ reported that first-order features exhibited the most reproducibility across various imaging parameters. Park s et al. ^24^ and Choe J et al. ^25^ reported that convolution network-based super resolution (SR) algorithms and kernel-converted images had reduced effects on the reproducibility of RF with variations in slice thickness and reconstruction kernels. Yang et al. ^26^ employed a resampling technique to standardize the voxel measurement of both thick and thin section CT images to 1x1x1 mm ^3^ using linear interpolation and observed that, following resampling of thicker images, 202 RF (66.2%, 202/305) exhibited a noteworthy reduction in variability of RF compared to the original non-resampled data ( Table 5).

**Table 5.** Comparison of reproducibility of radiomic features with CT technical parameters and Slice thickness combinations in lung cancer between current study and other recent studies.

Author name (year)	Our study (2023)	Lu et al. ^21^ (2016)	Erdal et al. ^20^ (2020)	Yang et al. ^26^ (2020)	Emaminejad et al. ^22^ (2021)
Pathology studied	Lung tumors (SCLC, NSCLCL)	Lung cancer	Lung nodules	Solid pulmonary nodules	Lung cancer
Study Procedure	CECT	NCCT	NCCT	CECT	NCCT
Technical parameters	ST (2 and 5-mm)	IRA (Lung and standard) ST	Dose levels (4) Kernels (10) Thicknesses (8)	ST (1.25 mm and 5 mm)	Dose levels (100 %, 50%, 25% and 10 %) ST (0.6, 1 and 2 mm) Reconstruction kernel (smooth, medium, sharp)
RF extracted	107	89	28	396	226
Features extracted	Shape, GLDM, GLCM First order, GLRLM GLSZM, NGTDM	Tumor size, Shape, Boundary shape, Sharpness, Density distributions with and without spatial information	Histogram, GLCM, RLM, NGDLM,NGTDM	Histogram, Geometry Texture features	First-order, Wavelet Features, GLDM, GLRLM, GLCM, GLSZM, NGTDM
Reproducibility of RF	Shape based features (57.1%), First order (44.4%) features showed highest reproducibility compared to other RF categories	Eight of the feature groups associated with dimensions, form, and rough texture exhibited consistent reproducibility across all combinations	Density feature was robust against dose changes, Skewness was robust for kernel and ST, Deviation was weakest feature for all cases. GLCM category was least reproducible	In non-resampled data, 239 features were shown significant differences between thin and thick slice. 66 RF were reproducible. In resampled data, 202 features exhibited significant differences between two thicknesses. 103 features were reproducible.	Seventeen and Eighteen features were reproducible with respect to dose and kernel changes. Only one to five features were reproducible with changes in slice thickness

For the GLRLM and GLSZM categories, reproducibility rates were 25% and 6.25 %, respectively, in the current study. A Study by Emaminejad et al. ^22^ similarly found that GLRLM Run length non uniformity (1 of 9 features) and GLSZM (1 of 10 features) displayed very limited reproducibility against the dose and kernel variations with varying slice thickness. Contrary to the study reported by Liu J et al. ^27^ which demonstrated that NGTDM exhibited good reproducibility, we did not observe any reproducible features in NGTDM. The reason for this disparity is attributed to differences in technical parameters, specifically in terms of dose variation, rather than slice thickness.

The study has few limitations. Firstly, the sample size was relatively small, as it is time bound study with prospective data collection of patients who underwent CT scan with histopathological proven cases of lung cancer. A larger sample size is required to confirm the reproducibility of RF with slice thickness. This may limit the generalizability of the findings to a broader population. Secondly, we did not analyze whether a thinner slice thickness would result in better performance of radiomic models for predicting lung cancer. Thirdly, a single image acquisition variable such as slice thickness was examined to determine how it affects the reproducibility of radiomic features. Additionally, the study focused on a specific CT scanner model (128-Incisive CT by Philips Health care), and the results might be informed by scanner-specific characteristics. Generalizing the findings to other CT scanners would require further investigation.

In terms of potential implications for clinical practice, the study underscores the importance of considering the slice thickness when utilizing radiomics in the assessment of lung tumors. Clinicians should be aware that variations in slice thickness can introduce significant variability in RF. This finding emphasizes the need for standardizing imaging protocols, particularly in the context of lung cancer diagnosis and treatment planning. The results also highlight the critical role of shape-based features in radiomics, as they demonstrated the highest reproducibility in this study. Clinicians incorporating radiomic analysis into their practice should be attentive to the choice of features, giving preference to those with higher reproducibility for more reliable and consistent results.

## Conclusion

Radiomics has the potential to transform lung cancer diagnosis, follow-up, and therapy planning by enabling individualised management in a non-invasive and an economical manner. Our study found that ST is the main parameter impacting the reproducibility of CT-RF for lung tumours. The original contribution of this study lies in its systematic examination of the influence of ST on the reproducibility of CT-RF in lung tumors. By identifying specific categories of RF that are more or less affected by variations in ST, the study provides valuable insights for researchers and clinicians working with radiomics in the field of lung cancer. This information could contribute to the refinement of imaging protocols, the standardization of radiomic analyses, and the interpretation of radiomic data in oncology. The study also increases awareness regarding the significance of accurately configuring imaging acquisition parameters in the context of radiomic/radio genomic approaches. Standardization of technical parameters and protocols is necessary when conducting multicentre studies, as these factors can impact the diagnostic performance of Machine Learning (ML) models developed using radiomic features.

## Data Availability

Figshare: F1000 Data Radiomic Features for 2-mm and 5-mm Slice thickness. https://doi.org/10.6084/m9.figshare.23935491. ^28^ This project contains the following underlying data:
- RF of 2 mm and 5mm ST (Spread Sheet) - CCC of RF (Spread Sheet) - Demographic characteristics of each patient F1000 (Spread Sheet) - CT images of all 32 patients (DICOM images) Data are available under the terms of the Creative Commons Attribution 4.0 International license (CC-BY 4.0).
