# Supplementary material for: Impact of slice thickness on reproducibility of CT radiomic features of lung tumors
Source: F1000Res. 2023 Dec 1;12:1319. Originally published 2023 Oct 12. [Version 2] doi: 10.12688/f1000research.141148.2 (PMC10918310; doi:10.12688/f1000research.141148.2)
Supplement: Peer review history

# Reviewer report: Reviewer response for version 2

**DOI:** 10.5256/f1000research.159376.r250915

**Published:** 2024-03-06

**Authors:** Tarun Gangil

**Affiliations:** 1 The institute of cancer research, London, UK

Major comments

 Authors should indicate the clinical validity of radiomics results. It is advised to authors to first specify the clinical relevance of variability of slice thickness, in the volumetric interpretation of the lung tumors. How the volumetric segmentation of 2mm slice thickness vary from the one with 5mm(without radiomics). Cite clinical research papers for the same. Further it can be added that radiomics features also supports the known clinical facts.

 Minor Comments

 Revision on Grammatical errors. 

 Suggestions to editor 

 It is an excellent research, where all the statistical process is well explained and can be reproduced, after addressing the major comment.

Is the work clearly and accurately presented and does it cite the current literature?

Yes

If applicable, is the statistical analysis and its interpretation appropriate?

Yes

Are all the source data underlying the results available to ensure full reproducibility?

Yes

Is the study design appropriate and is the work technically sound?

Yes

Are the conclusions drawn adequately supported by the results?

Yes

Are sufficient details of methods and analysis provided to allow replication by others?

Yes

Reviewer Expertise:

Deep learning, Image Processing, Machine Learning

I confirm that I have read this submission and believe that I have an appropriate level of expertise to confirm that it is of an acceptable scientific standard.

# Reviewer report: Reviewer response for version 2

**DOI:** 10.5256/f1000research.159376.r227714

**Published:** 2023-12-11

**Authors:** Mubarak Taiwo Mustapha

**Affiliations:** 1 Near East University, TRNC Mersin, Nicosia, Turkey

I can confirm that all my comments and suggestions have been addressed.

Is the work clearly and accurately presented and does it cite the current literature?

Yes

If applicable, is the statistical analysis and its interpretation appropriate?

Yes

Are all the source data underlying the results available to ensure full reproducibility?

Yes

Is the study design appropriate and is the work technically sound?

Partly

Are the conclusions drawn adequately supported by the results?

Yes

Are sufficient details of methods and analysis provided to allow replication by others?

Partly

Reviewer Expertise:

NA

I confirm that I have read this submission and believe that I have an appropriate level of expertise to confirm that it is of an acceptable scientific standard.

# Reviewer report: Reviewer response for version 1

**DOI:** 10.5256/f1000research.154563.r214642

**Published:** 2023-10-26

**Authors:** Mubarak Taiwo Mustapha

**Affiliations:** 1 Near East University, TRNC Mersin, Nicosia, Turkey

Major comments

1. The abstract could benefit from a more explicit mention of the study's original contribution to the field.
2. Directly state the main research question that the study addresses.
3. Include more details about the image acquisition parameters (e.g., exposure factors, acquisition protocols) to ensure reproducibility of the study.
4. Explicitly address the limitations of the study, as well as potential implications for clinical practice.

Minor comments

1. In the methods section, provide a brief rationale for excluding patients with ground glass nodules (GGN) and lesions measuring less than 4mm.
2. In the Methods section, the date of approval from the Institutional Ethical Committee (12th August 2022) and the CTRI registration date (15th September 2022) are provided. It might be helpful to briefly explain the time gap between these dates, if relevant.
3. In the Results section, please clarify what RF stands for upon first mention. While it's explained later in the text, an initial definition or expansion would be helpful for reader clarity.
4. In the Discussion section, while comparing the study's findings with previous research, consider mentioning any discrepancies or agreement with other studies in terms of reproducibility outcomes.
5. The Conclusion section succinctly summarizes the main findings and emphasizes the importance of standardizing technical parameters. It could further benefit from a statement about the study's potential impact on clinical practice or future research directions.
6. In the Conclusion, it might be worth reiterating the study's original contribution to the field, especially in terms of its focus on slice thickness and reproducibility of radiomic features.

Is the work clearly and accurately presented and does it cite the current literature?

Yes

If applicable, is the statistical analysis and its interpretation appropriate?

Yes

Are all the source data underlying the results available to ensure full reproducibility?

Yes

Is the study design appropriate and is the work technically sound?

Partly

Are the conclusions drawn adequately supported by the results?

Yes

Are sufficient details of methods and analysis provided to allow replication by others?

Partly

Reviewer Expertise:

Medical imaging; Artificial intelligence;

I confirm that I have read this submission and believe that I have an appropriate level of expertise to confirm that it is of an acceptable scientific standard, however I have significant reservations, as outlined above.

# Response

**Published:** 2023-11-27

**Authors:** Saikiran Pendem

**Affiliations:** Medical Imaging Technology, Manipal Academy of Higher Education, Manipal, Karnataka, India

**Major Comments**

**1. The abstract could benefit from a more explicit mention of the study's original contribution to the field.**

**Response: **The present study helps in identifying the specific Radiomic Feature (RF) categories affected by variations in Slice thickness (ST) and provides valuable insights for researchers and clinicians working with RF in the field of Lung cancer (LC)

**2. Directly state the main research question that the study addresses.**

**Response: **As per the suggestion, the research gap is addressed in the introduction as below

 The research gap of the study centers on the application of radiomics in lung cancer, emphasizing the need for a deeper understanding of the reproducibility of radiomic features (RF) in the context of lung tumors.

**3. Include more details about the image acquisition parameters (e.g., exposure factors, acquisition protocols) to ensure reproducibility of the study**

**Response: **As per the suggestion, additional image acquisition parameters ( esposure factors such as kVp and mAs were already mentioned in Table 2) were included for ensuring the reproducibility of the study as below 

 Reconstruction algorithm - iDose ^4^, Level 3

 Spatial resolution-0.33

 Threshold (HU)- 150

 Window Width (WW)-400

 Window Level (WL)- 40

**4 Explicitly address the limitations of the study, as well as potential implications for clinical practice**

 As per the suggestion the limitations of the study, as well as potential implications for clinical practice were addressed in the discussion as below.

**Response: **The study has few limitations. Firstly, the sample size was relatively small, as it is time bound study with prospective data collection of patients who underwent CT scan with histopathological proven cases of lung cancer. A larger sample size is required to confirm the reproducibility of RF with slice thickness. This may limit the generalizability of the findings to a broader population. Secondly, we did not analyze whether a thinner slice thickness would result in better performance of radiomic models for predicting lung cancer. Thirdly, a single image acquisition variable such as slice thickness was examined to determine how it affects the reproducibility of radiomic features. Additionally, the study focused on a specific CT scanner model (128-Incisive CT by Philips Health care), and the results might be informed by scanner-specific characteristics. Generalizing the findings to other CT scanners would require further investigation.

 In terms of potential implications for clinical practice, the study underscores the importance of considering the slice thickness when utilizing radiomics in the assessment of lung tumors. Clinicians should be aware that variations in slice thickness can introduce significant variability in RF. This finding emphasizes the need for standardizing imaging protocols, particularly in the context of lung cancer diagnosis and treatment planning. The results also highlight the critical role of shape-based features in radiomics, as they demonstrated the highest reproducibility in this study. Clinicians incorporating radiomic analysis into their practice should be attentive to the choice of features, giving preference to those with higher reproducibility for more reliable and consistent results.

**Minor comments**

**5. In the methods section, provide a brief rationale for excluding patients with ground glass nodules (GGN) and lesions measuring less than 4mm.**

**Response: **Ground glass nodules (GGN) often represent a distinct category of pulmonary nodules that may differ from solid lesions. Hence they were excluded to focus on a more homogenous sample. Limiting the inclusion criteria to lesions measuring at least 4mm ensures a more consistent and reliable measurement. Smaller lesions might present challenges in terms of accurate radiomic feature extraction and may be subject to greater variability due to partial volume effects, potentially influencing the study’s reproducibility findings.

**6.In the Methods section, the date of approval from the Institutional Ethical Committee (12th August 2022) and the CTRI registration date (15th September 2022) are provided. It might be helpful to briefly explain the time gap between these dates, if relevant.**

**Response: **The CTRI registration will be done after receiving the Institutional ethical committee approval letter. Hence it took a month time for completing the CTRI registration.

**7.In the Results section, please clarify what RF stands for upon first mention. While it's explained later in the text, an initial definition or expansion would be helpful for reader clarity.**

**Response: **As per the suggestions, the Radiomic features (RF) abbreviation is provided in the results section.

**8.In the Discussion section, while comparing the study's findings with previous research, consider mentioning any discrepancies or agreement with other studies in terms of reproducibility outcomes.**

**Response: **The study findings with previous research were already discussed in discussion section and Table 5. Discrepancies and agreement with other studies in terms of reproducibility outcomes were also discussed and provided in Table 5

**9.The Conclusion section succinctly summarizes the main findings and emphasizes the importance of standardizing technical parameters. It could further benefit from a statement about the study's potential impact on clinical practice or future research directions.**

**Response: **Our study information could contribute to the refinement of imaging protocols, the standardization of radiomic analyses, and the interpretation of radiomic data in oncology. (included the same in conclusion)

**10. In the Conclusion, it might be worth reiterating the study's original contribution to the field, especially in terms of its focus on slice thickness and reproducibility of radiomic features**

**Response: **The original contribution of this study lies in its systematic examination of the influence of slice thickness on the reproducibility of CT-RF in lung tumors. By identifying specific categories of radiomic features that are more or less affected by variations in slice thickness, the study provides valuable insights for researchers and clinicians working with radiomics in the field of lung cancer. (Included the same in conclusion)
